# Supplementary material for: Genetic determinants of monocyte splicing are enriched for disease susceptibility loci
Source: Nat Commun. 2025 Sep 29;16:8616. doi: 10.1038/s41467-025-63624-7 (PMC12480492; doi:10.1038/s41467-025-63624-7)
Supplement: Supplementary file 1 — Supplementary Information [file 41467_2025_63624_MOESM1_ESM.pdf]

Supplementary Information

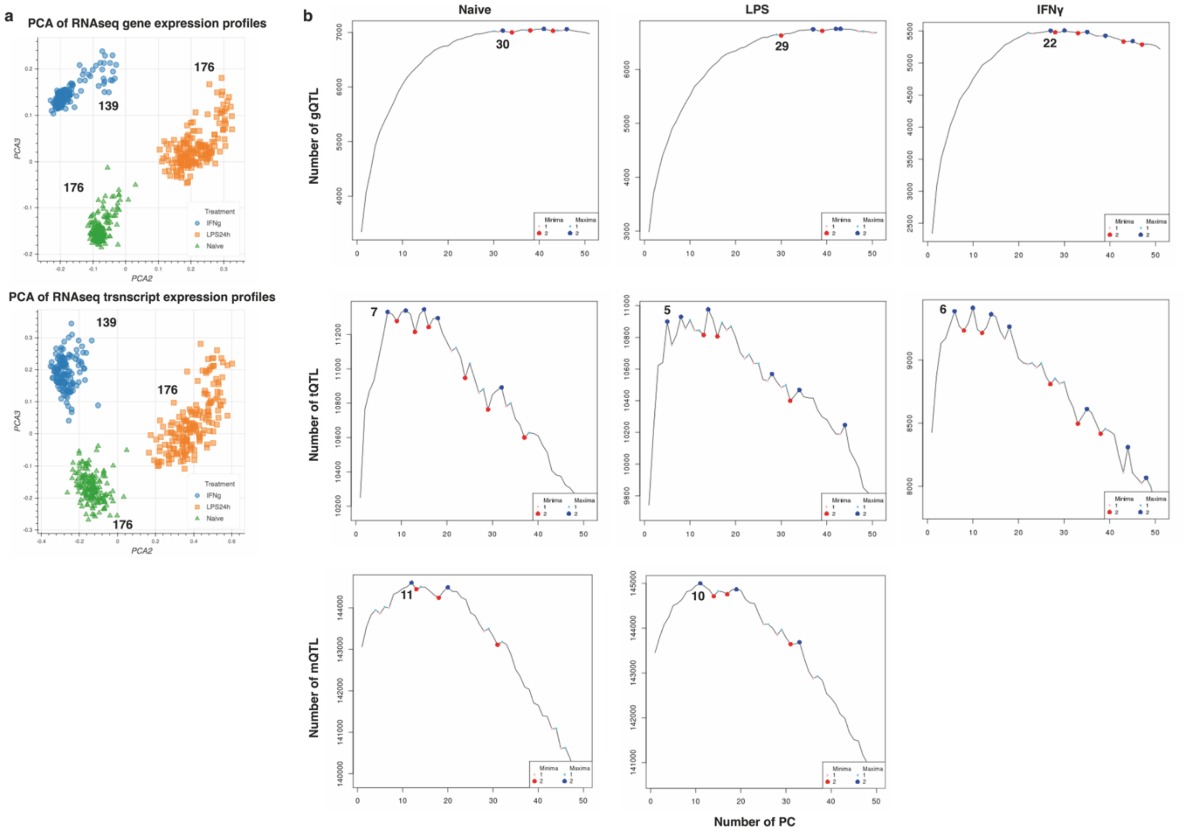

Supplementary figure S1. PCA of gene expression and selection of principal components for eQTL mapping. (A) Principal component analysis (PCA) using gene and transcript expression profiling from high-throughput RNA sequencing (RNA-seq) showed that samples were clearly divided into different groups for naïve and situmulated monocytes. (B) Permutations were used to test profiles of gene, transcript, and methylation level for a range of zero to 50 PCs. Inflection and local maxima concepts guided the inclusion of the dominant PC as a covariate. Our objective was to select the optimal number of PCs to use as covariates in eQTL analysis, resulting in the maximum number of eQTLs and correcting for confounding factors.

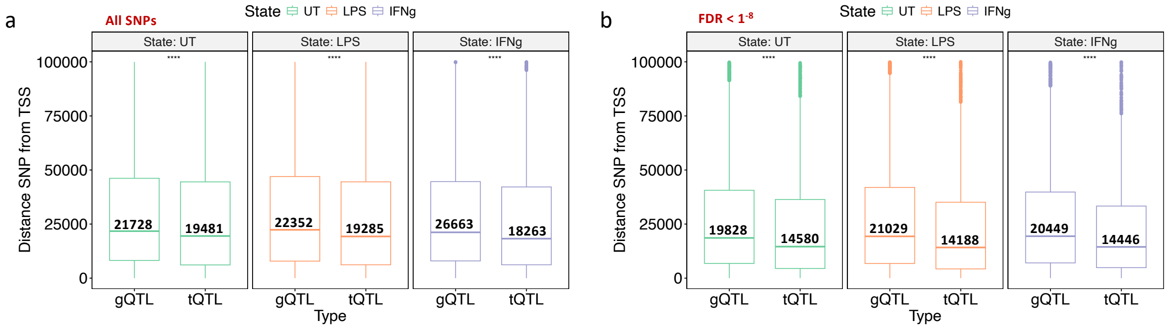

Supplementary figure S2. Difference between the distance of best-hit tSNPs from transcription start sites (TSS) in comparison with gSNPs in naïve and stimulated monocytes (±100kb, FDR < 0.01). (a) Using g/tQTL FDR < 0.01, we evaluated the distribution of tSNPs and gSNPs in relation to TSS. Our study showed that tSNPs were more closely related to TSS than gSNPs, which suggests a more concentrated regulatory influence for tSNPs. The finding is in line with the belief that regulatory regions closer to TSS are richer in tSNPs. (b) The proximity of tSNPs to TSS in naïve and IFN-γ monocytes (g/tQTL FDR < $1\text{x}10^{-9}$) was greater than that of gSNPs, which suggests a more local regulatory influence. The findings shed light on how gene and transcript regulatory mechanisms respond dynamically to cellular stimuli. Stars are used to indicate the statistical significance of the difference between gQTL and tQTL in a boxplot. These stars represent the p-value obtained from the t-test. A p-value lower than 1^{-4} indicates a very high level of significance, which is represented by four stars.

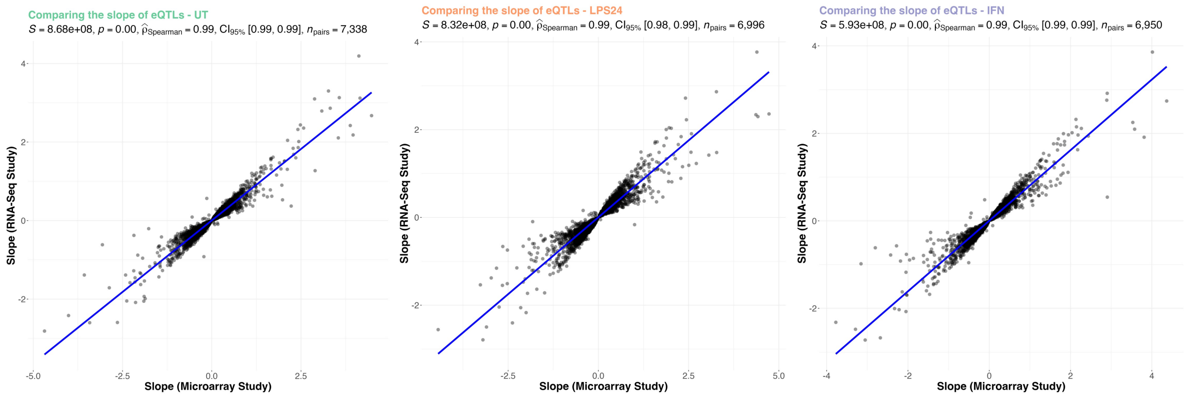

Supplementary figure S3. The regulation effect size, which is linearly and normally distributed, is observed across microarray and RNA-Seq studies, as reflected by the Spearman nonparametric correlation (Shapiro-Wilk normality test p-value < 0.001).

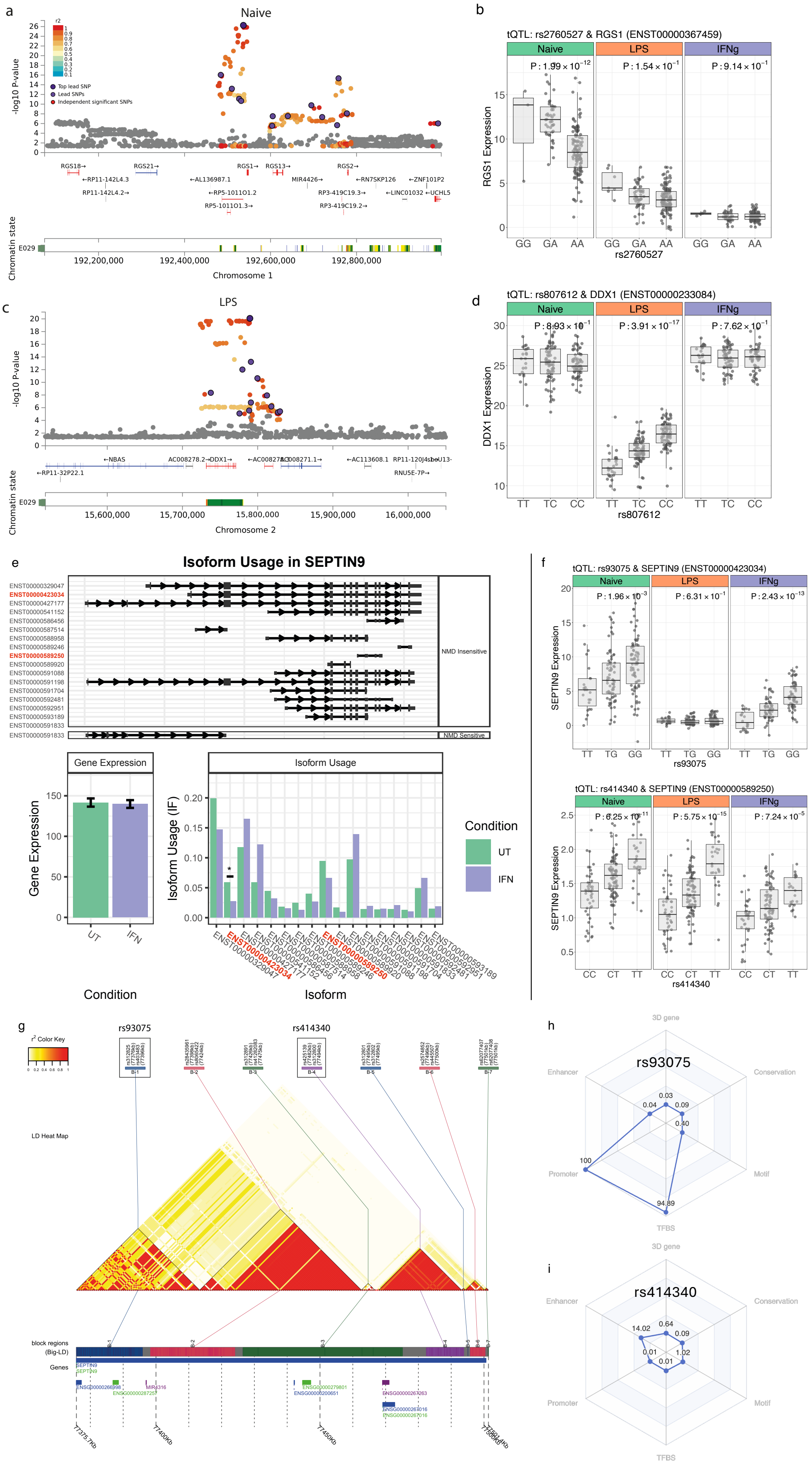

a
Naive
-log10 P-value
Top lead SNP
Lead SNPs
Independent significant SNPs
Chromatin state
Chromosome 1
b
tQTL: rs2760527 & RGS1 (ENST00000367459)
Naive
LPS
IFNg
RGS1 Expression
rs2760527
c
LPS
Chromosome 2
d
tQTL: rs807612 & DDX1 (ENST00000233084)
DDX1 Expression
rs807612
e
Isoform Usage in SEPTIN9
NMD Insensitive
NMD Sensitive
Gene Expression
Isoform Usage
Isoform Usage (IF)
Condition
UT
IFN
Isoform
f
tQTL: rs93075 & SEPTIN9 (ENST00000423034)
SEPTIN9 Expression
rs93075
tQTL: rs414340 & SEPTIN9 (ENST00000589250)
rs414340
g
rs93075
rs414340
LD Heat Map
block regions (Big-LD)
Genes
h
rs93075
3D gene
Conservation
Motif
TFBS
Promoter
Enhancer
i
rs414340

Supplementary figure S4. Example of genetic determinants of condition-specific tQTLs. (a) Read coverage plot of a transcript (ENST00000367459) of negative regulator of G-protein signalling 1 (*RGS1*) across all the samples. (b) Local association plots for tQTL between the rs2760527 genetic variant and ENST00000367459 in naïve monocyte cells. SNP rs2760527 has a loss of function impact on the *LEF1* transcription factor binding sequence motif ($p < 0.001$)[129]. We found rs2760527, in R2 and D' of 1 with rs2816316, a GWAS risk allele for celiac disease[130]. (c) Read coverage plot of a transcript of DDX1 (ENST00000233084) across all the samples. DDX1 is an RNA helicase protein that functions to promote RNA splicing, DNA recombination, and induce proinflammatory cytokines[131, 132, 133]. (d) Local association plots for context-specific tQTL between the rs807612 variant and expression of the ENST00000233084 transcript after LPS stimulation. (e) switchPlot presents a significant switch in the isoform usage of *SEPTIN9* in response to IFN-γ including gene expression and isoform fraction (IF) values. IF quantifies the fraction of a mean isoform expression and a mean gene expression. Highlighted isoforms (red colour) showing evidence of tQTLs after exposure to IFN-γ. *SEPTIN9* encodes a gene involved in cytokinesis[134]. (f) Local association plots for tQTL between the rs93075 and rs414340 genetic variants and ENST00000423034 and ENST00000589250 transcripts, respectively. Exposure to IFN-γ does not induce *SEPTIN9* gene expression but leads to distinct tQTL to separate transcripts involving separate regulatory variants. rs93075 and rs414340 are located within a CTCF binding site (ENSR00000565050) and an open chromatin region (ENSR00000098752), respectively. (g) The LD block heatmap displays the boundaries of the LD blocks, the first and last SNPs in each block, and gene information in the region (chr17:77375740-77501356, including 352 SNPs). (h-i) The functional significance score was applied to evaluate the functional significance of indicated SNPs in 6 categories[111]. The x axis of the boxplot represents the different alleles of the SNP that are related to the expression. The x axis of the boxplot represents the different alleles of the SNP that are related to the expression. Each box indicates a particular SNP allele, and the y-axis displays the expression levels of individuals with that allele. The normalized read count displays the expression levels of genes. The Fragments Per Kilobase Million (FPKM) metric displays the expression levels of transcripts. The 25th and 75th percentiles are represented in the box on the bottom and top. The box has a line that indicates the median of expression. The whiskers cover both the minimum and maximum values, with the exception of outliers. The sample size to generate box plots for naïve, LPS, and IFN- treated monocytes is 176, 176, and 138 respectively.

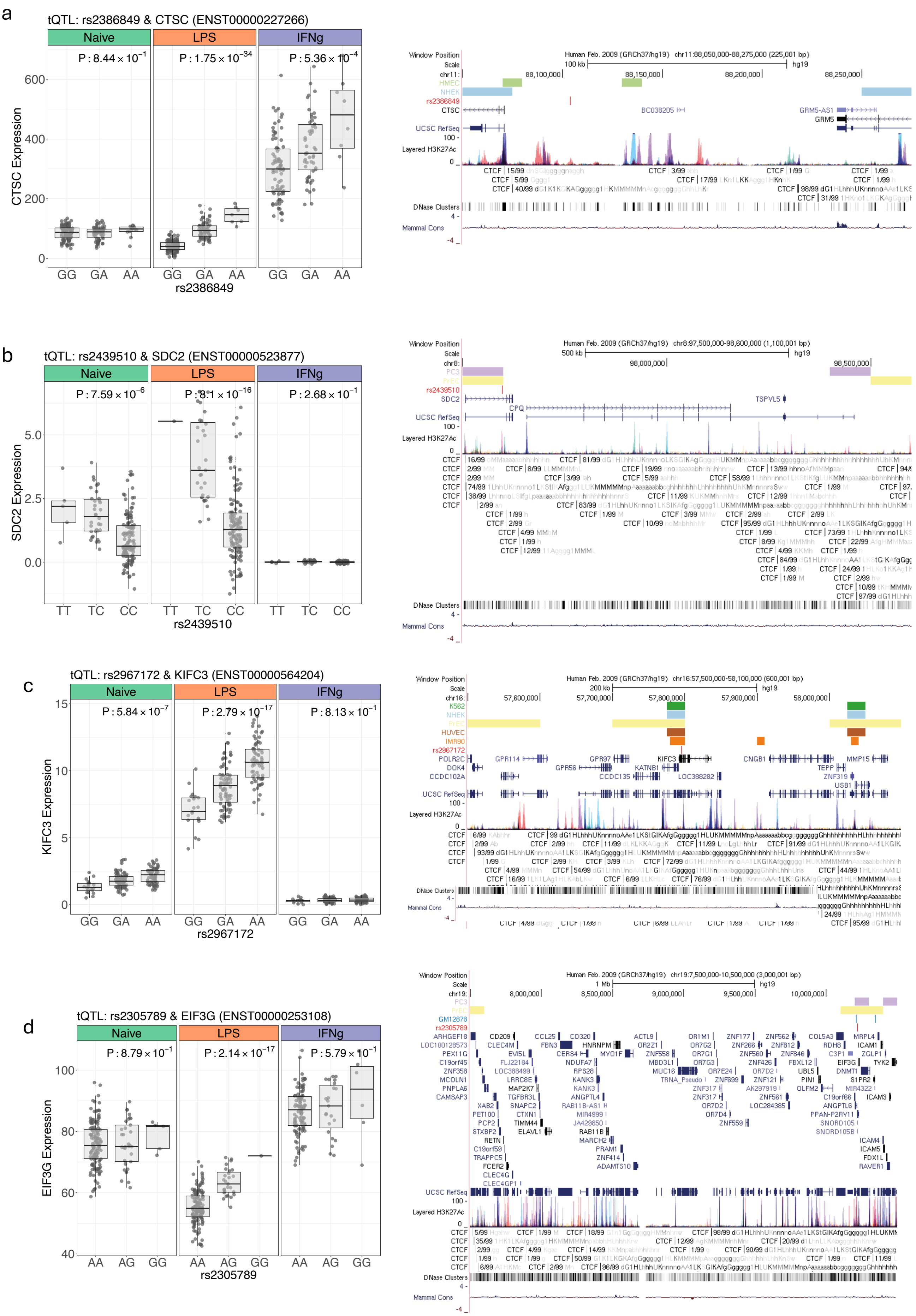

a
tQTL: rs2386849 & CTSC (ENST00000227266)
Naive
LPS
IFNg
P : 8.44 × 10^-1
P : 1.75 × 10^-34
P : 5.36 × 10^-4
CTSC Expression
GG GA AA
rs2386849
Window Position
Human Feb. 2009 (GRCh37/hg19) chr11:88,050,000-88,275,000 (225,001 bp)
Layered H3K27Ac
DNase Clusters
Mammal Cons
b
tQTL: rs2439510 & SDC2 (ENST00000523877)
Naive
LPS
IFNg
P : 7.59 × 10^-6
P : 8.1 × 10^-16
P : 2.68 × 10^-1
SDC2 Expression
TT TC CC
rs2439510
Window Position
Human Feb. 2009 (GRCh37/hg19) chr8:97,500,000-98,600,000 (1,100,001 bp)
Layered H3K27Ac
DNase Clusters
Mammal Cons
c
tQTL: rs2967172 & KIFC3 (ENST00000564204)
Naive
LPS
IFNg
P : 5.84 × 10^-7
P : 2.79 × 10^-17
P : 8.13 × 10^-1
KIFC3 Expression
GG GA AA
rs2967172
Window Position
Human Feb. 2009 (GRCh37/hg19) chr16:57,500,000-58,100,000 (600,001 bp)
Layered H3K27Ac
DNase Clusters
Mammal Cons
d
tQTL: rs2305789 & EIF3G (ENST00000253108)
Naive
LPS
IFNg
P : 8.79 × 10^-1
P : 2.14 × 10^-17
P : 5.79 × 10^-1
EIF3G Expression
AA AG GG
rs2305789
Window Position
Human Feb. 2009 (GRCh37/hg19) chr19:7,500,000-10,500,000 (3,000,001 bp)
Layered H3K27Ac
DNase Clusters
Mammal Cons

Supplementary figure S5. Monocyte stimulus-specific cis-tQTLs post LPS and IFN-γ treatments. Local association and genome browser plots for (**a**) context-specific tQTLs between the rs2386849 variant and the expression of *CTSC* transcript (ENST00000227266) after LPS and IFN-γ treatments. CTSC (Cathepsin C) is a central coordinator of proteinases in the immune system cells. (**b**) Context-specific tQTLs between the rs2439510 variant and the expression of *SDC2* transcript (ENST00000523877) after LPS treatment. *SDC2* is a gene that codes for transmembrane proteins and is linked to cell proliferation, migration, and interactions between cells and the matrix. (**c**) Context-specific tQTLs between the rs2967172 variant and the expression of *KIFC3* transcript (ENST00000564204) in LPS-treated and naïve monocytes. KIFC3 plays a role in the final stages of cell division. (**d**) context-specific tQTLs between the rs2305789 variant and the expression of *EIF3G* transcript (ENST00000253108) in LPS-treated monocytes. EIF3G is a component that bonds to RNA in the eukaryotic translation initiation factor 3 (eIF-3) complex. To allow comparison with the output of the regression model the optimal number of P.C. used to regress out expression changes attributable to the effect of the non-genetic covariates in local association plots. The x axis of the boxplot represents the different alleles of the SNP that are related to the expression. Each box indicates a particular SNP allele, and the y-axis displays the expression levels of individuals with that allele. The normalized read count displays the expression levels of genes. The Fragments Per Kilobase Million (FPKM) metric displays the expression levels of transcripts. The 25th and 75th percentiles are represented in the box on the bottom and top. The box has a line that indicates the median of expression. The whiskers cover both the minimum and maximum values, with the exception of outliers. The sample size to generate box plots for naïve, LPS, and IFN- treated monocytes is 176, 176, and 138 respectively.

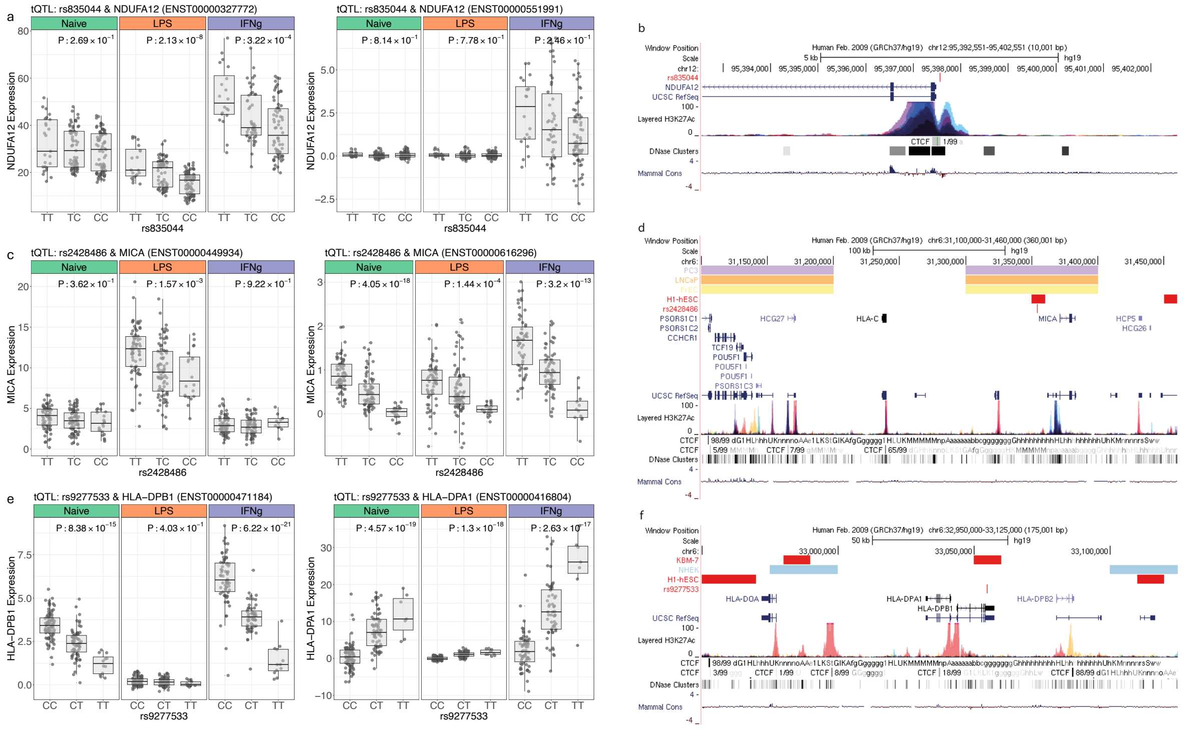

Supplementary figure S6. Examples of gene variants associated with the different isoforms of a target gene including different transcripts but with different directions. For *NDUFA12* and *MICA* tQTL genes, we found independent SNPs that were associated with different isoforms of the target gene in a context-specific manner (a-d). For the *HLA–DPB1* tQTL gene, the effect of rs9277533 alleles was revealed on IFN-γ and naïve conditions for two different transcripts but with different directions (e-f). To allow comparison with the output of the regression model the optimal number of P.C. used to regress out expression changes attributable to the effect of the non-genetic covariates in local association plots. The x axis of the boxplot represents the different alleles of the SNP that are related to the expression. Each box indicates a particular SNP allele, and the y-axis displays the expression levels of individuals with that allele. The normalized read count displays the expression levels of genes. The Fragments Per Kilobase Million (FPKM) metric displays the expression levels of transcripts. The 25th and 75th percentiles are represented in the box on the bottom and top. The box has a line that indicates the median of expression. The whiskers cover both the minimum and maximum values, with the exception of outliers. The sample size to generate box plots for naïve, LPS, and IFN- treated monocytes is 176, 176, and 138 respectively.

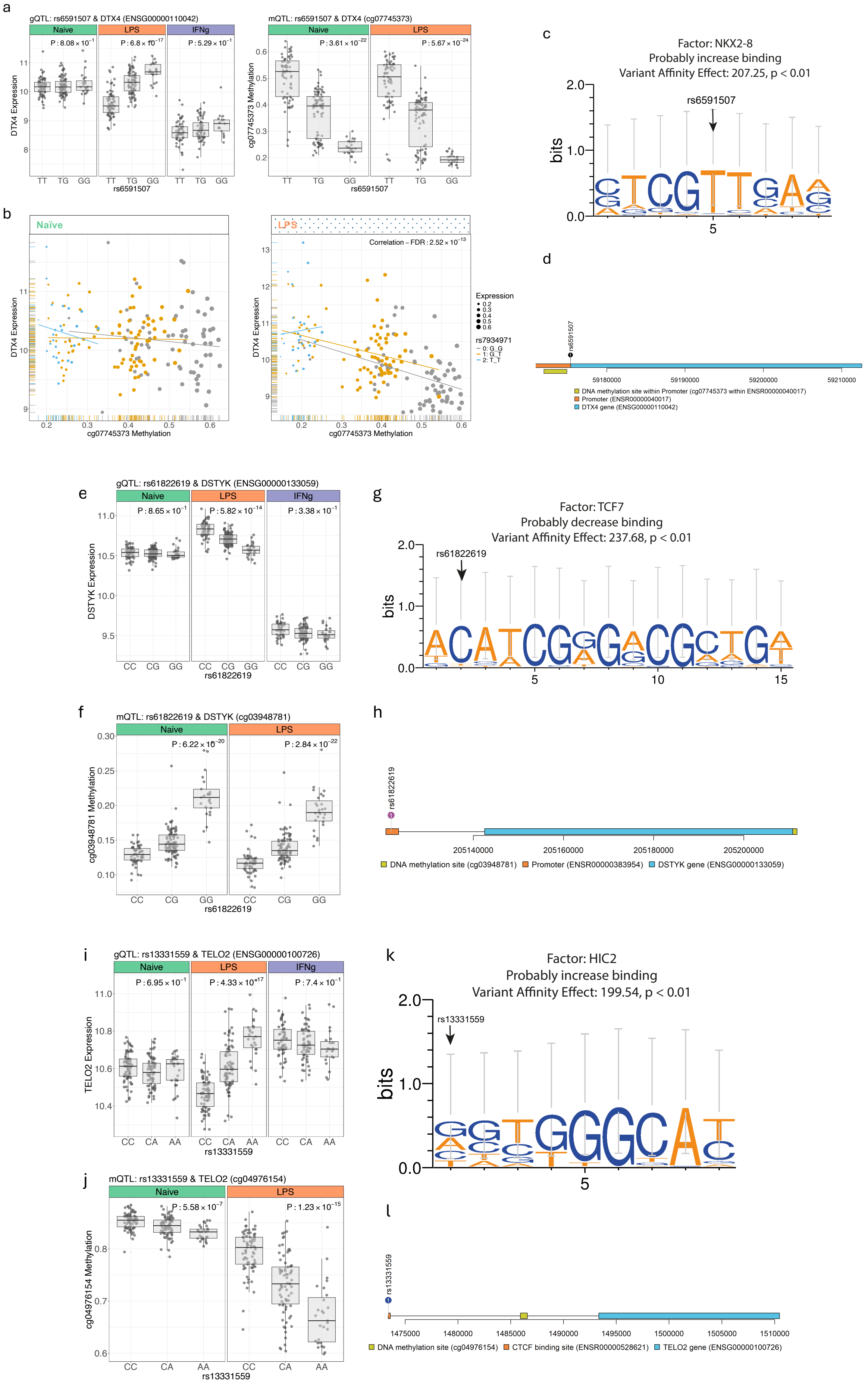

a
gQTL: rs6591507 & DTX4 (ENSG00000110042)
mQTL: rs6591507 & DTX4 (cg07745373)
Naive
LPS
IFNg
DTX4 Expression
cg07745373 Methylation
rs6591507
b
Naïve
LPS
Correlation - FDR: 2.52 x 10^-13
DTX4 Expression
cg07745373 Methylation
Expression
rs7934971
c
Factor: NKX2-8
Probably increase binding
Variant Affinity Effect: 207.25, p < 0.01
rs6591507
bits
d
DNA methylation site within Promoter (cg07745373 within ENSR00000040017)
Promoter (ENSR00000040017)
DTX4 gene (ENSG00000110042)
e
gQTL: rs61822619 & DSTYK (ENSG00000133059)
DSTYK Expression
rs61822619
f
mQTL: rs61822619 & DSTYK (cg03948781)
cg03948781 Methylation
g
Factor: TCF7
Probably decrease binding
Variant Affinity Effect: 237.68, p < 0.01
rs61822619
h
DNA methylation site (cg03948781)
Promoter (ENSR00000383954)
DSTYK gene (ENSG00000133059)
i
gQTL: rs13331559 & TELO2 (ENSG00000100726)
TELO2 Expression
rs13331559
j
mQTL: rs13331559 & TELO2 (cg04976154)
cg04976154 Methylation
k
Factor: HIC2
Probably increase binding
Variant Affinity Effect: 199.54, p < 0.01
rs13331559
l
DNA methylation site (cg04976154)
CTCF binding site (ENSR00000528621)
TELO2 gene (ENSG00000100726)

Supplementary figure S7. More examples of interactions between DNA methylation and expression. (a) A promoter genetic variant (rs6591507) influences the methylation level (cg07745373) and stimulation-specific gene expression (*DTX4*). A high probability of shared causal effects was suggested by the PPH4 values for expression and methylation being 1. eQTL significantly affects the expression of *DTX4* in LPS-stimulated monocytes. *DTX4* (Deltex E3 Ubiquitin Ligase 4) is a cytosolic sensor of pathogen-associated DNA and is part of an inducible protein complex that is induced in response to viral infection. (b) The expression of *DTX4* correlates with the methylation level of cg07745373 in LPS-stimulated monocytes. The expression of *DTX4* and cg07745373 is coloured by the genotype SNP rs6591507. (c) The genomic sequence surrounding SNP rs6591507 is aligned to a logo graphic representing the consensus binding site of the NKX2-8 transcription factor. NKX2-8 (NK2 Homeobox 8) is a developmental regulator containing a homeobox that is associated with DNA-binding transcription factor activity. (d) A promoter polymorphism influences the stimulation-specific *DSTYK* expression and methylation site within the same regulatory region. A high probability of shared causal effects was suggested by the PPH4 values for expression and methylation being 1. (e-f) A promoter genetic variant (rs61822619) influences the methylation level (cg03948781) and stimulation-specific gene expression (*DSTYK*). *DSTYK*, also known as Dual Serine/Threonine and Tyrosine Protein Kinase, is a positive regulator of ERK phosphorylation and is involved in regulating both caspase-dependent and caspase-independent apoptosis. (g) The genomic sequence surrounding SNP rs61822619 is aligned to a logo graphic representing the consensus binding site of TCF7 (T-Cell-Specific Transcription Factor 1) which encodes an enhancer-binding factor of high mobility group (HMG) box transcriptional activators. (h) A promoter polymorphism with regulatory activity influences the expression of stimulation-specific DSTYK and the methylation site downstream of the regulatory region. (i-j) An CTCF binding site genetic variant (rs13331559) influences the methylation level (cg04976154) and stimulation-specific gene expression (TELO2). TELO2 (telomere maintenance 2) is associated with the mTOR signalling pathway (KEGG) and Fanconi anemia disease. A high probability of shared causal effects was suggested by the PPH4 values for expression and methylation being 1. (k) The genomic sequence surrounding SNP rs13331559 is aligned with a logo graphic representing the consensus binding site of HIC2 (Hypermethylated in Cancer 2 Protein). (l) An CTCF binding site influences the regulatory activity and stimulation-specific expression of TELO2 and its methylation site.

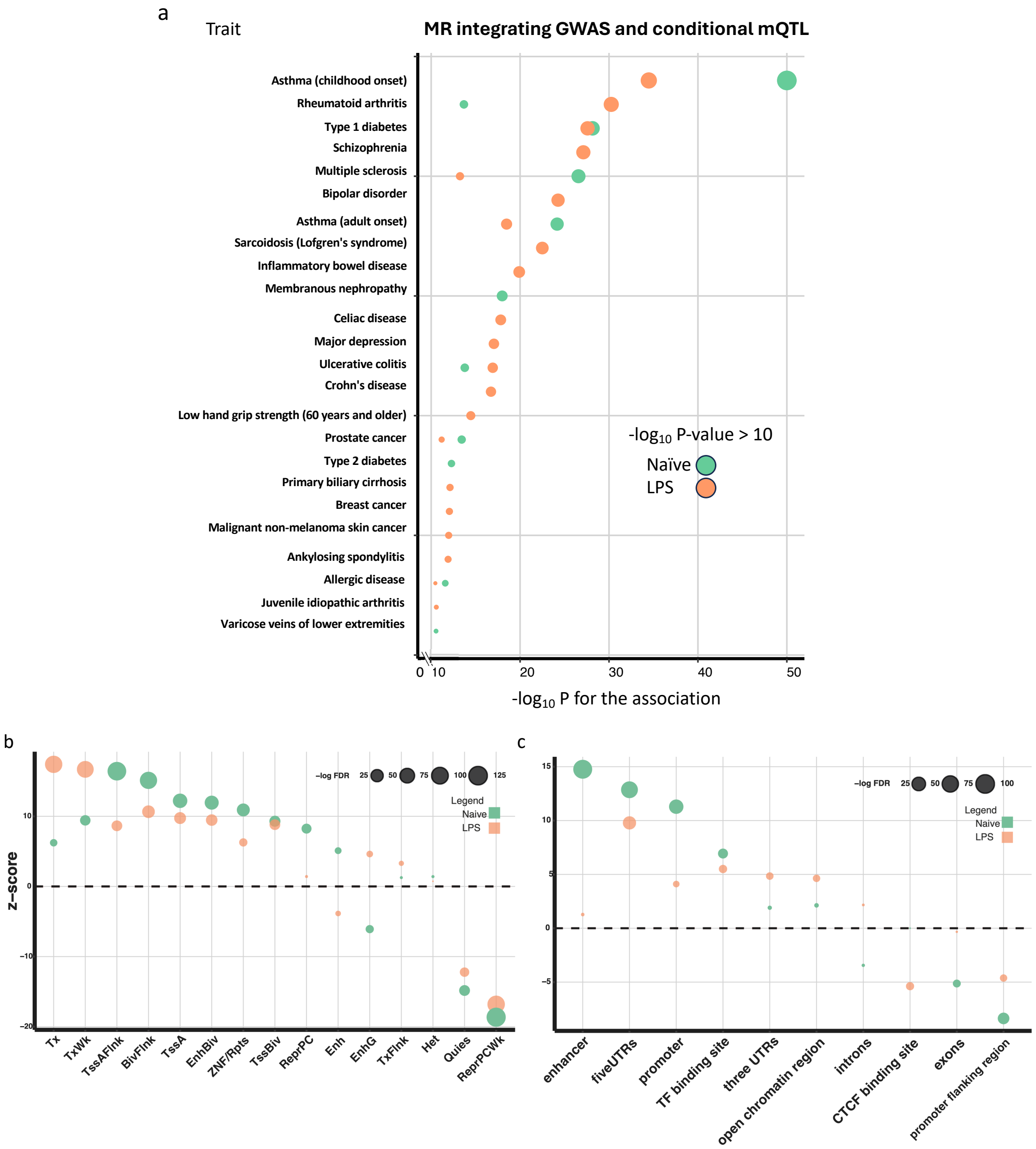

Supplementary figure S8. Enrichment of mSNPs with GWAS hist, chromatin and functional regions. (a) Analysis of causal relationships between traits and mSNPs specific to naïve and LPS contexts using GWAS summary statistics and Mendelian randomization. (b) Functional annotations of mSNPs specific to naïve and LPS contexts using genomic features. The plot displays the z-score calculated using the proportion of mSNPs which have corresponding functional annotations assigned by biomaRt and UCSC databases in comparison to background SNP. (c) The enrichment of mSNPs specific to LPS context with chromatin state (E029 chromatin states dataset provided by the Roadmap Epigenomics Consortium) across primary monocytes from peripheral blood tissue. Abbreviations: TssAFlnk: Active TSS Flank, TxFlnk: Transcription, Tx: Transcription Flank, TxWk: Weak Transcription, EnhG: Gene Enhancer, Enh: Enhancer, ZNF/Rpts: Zinc Finger/Repeats, Het: Heterochromatin, TssBiv: Bivalent TSS, BivFlnk: Bivalent Flank, EnhBiv: Bivalent Enhancer, ReprPC:

Repressed Polycomb-repressed Complex, ReprPCWk: Weak Repressed Polycomb-repressed Complex, Quies: Quiescent chromatin. fiveUTRs: 5' Untranslated Regions. threeUTRs: 3' Untranslated Regions.

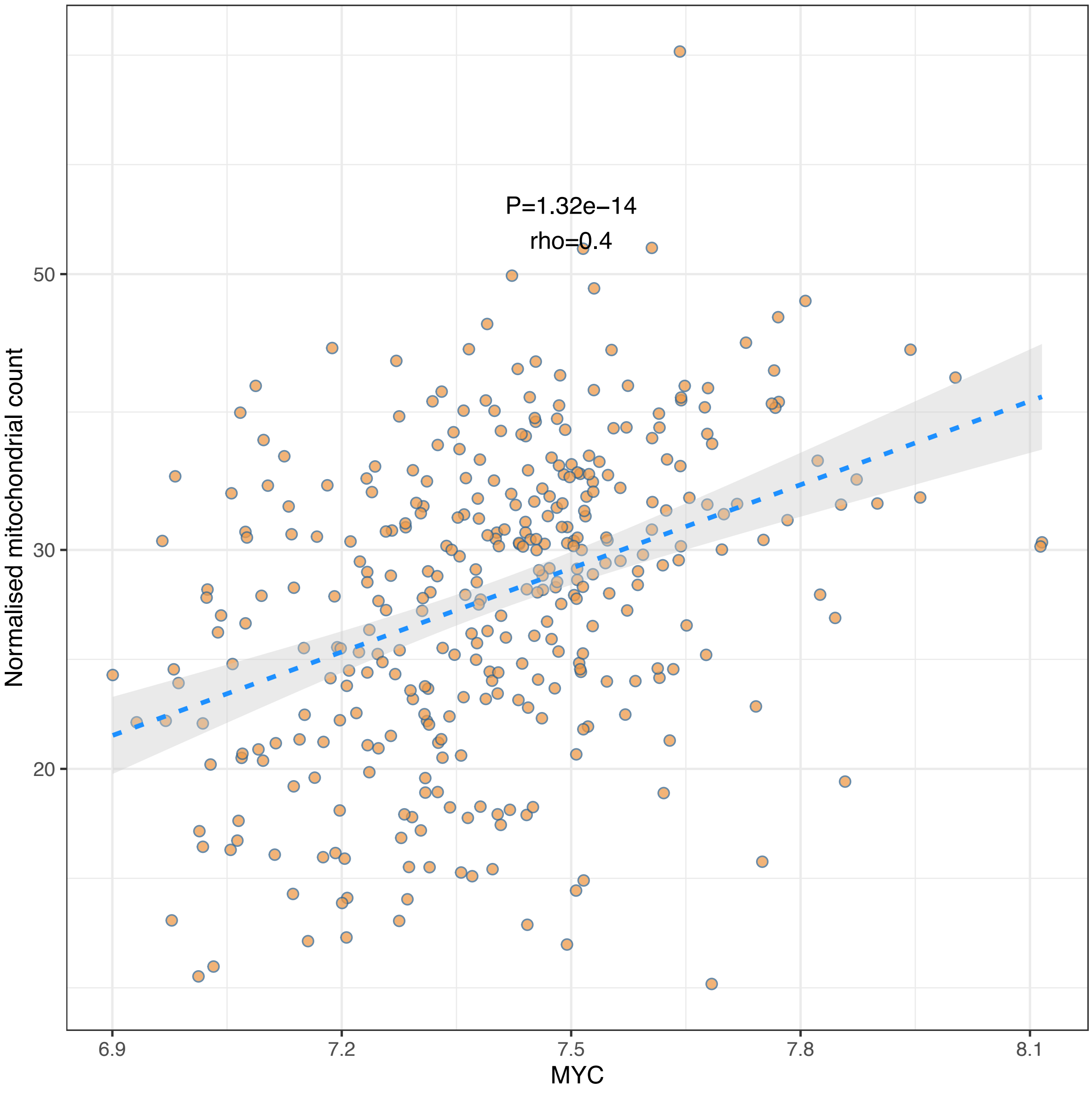

Supplementary figure S9. The mitochondrial count is highly correlated with MYC expression after IFN-γ treatment.

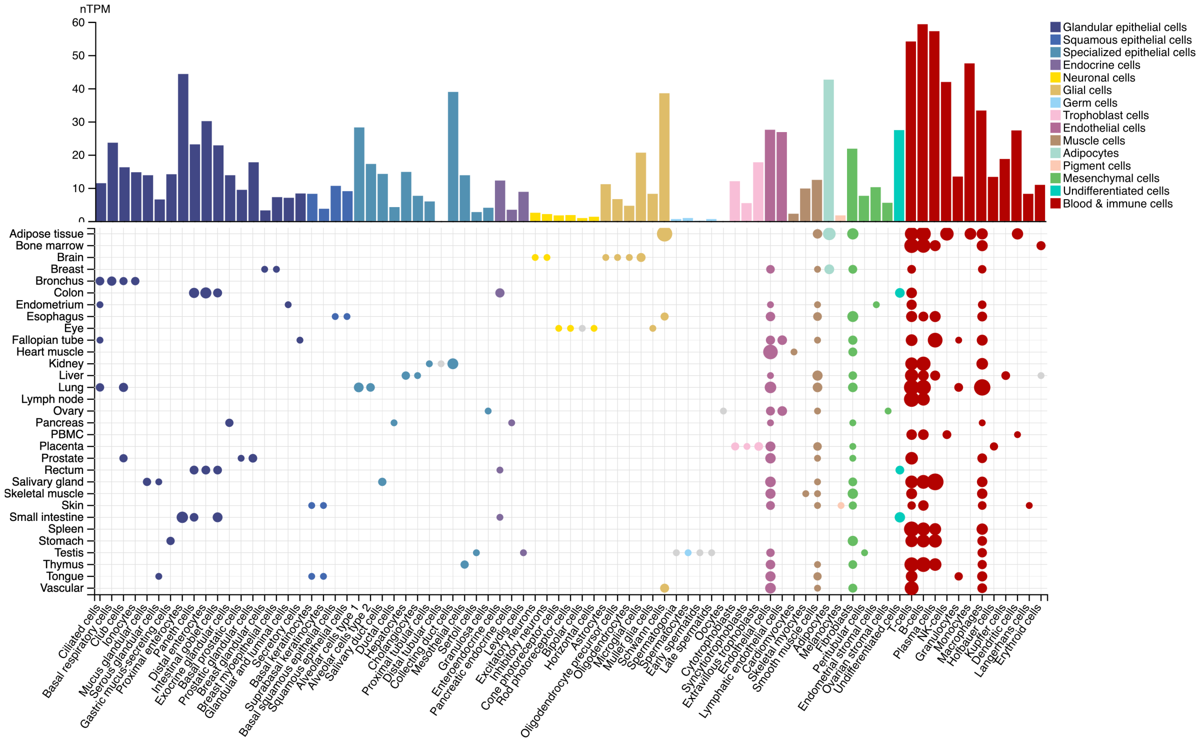

Supplementary figure S10. A condensed overview of the normalized single-cell RNA (nTPM) measurements for *ERAP2* in all human cell types. Color-coding is based on the grouping of cell types that share functional characteristics. Image adapted from the Human Protein Atlas (https://www.proteinatlas.org/)[135].

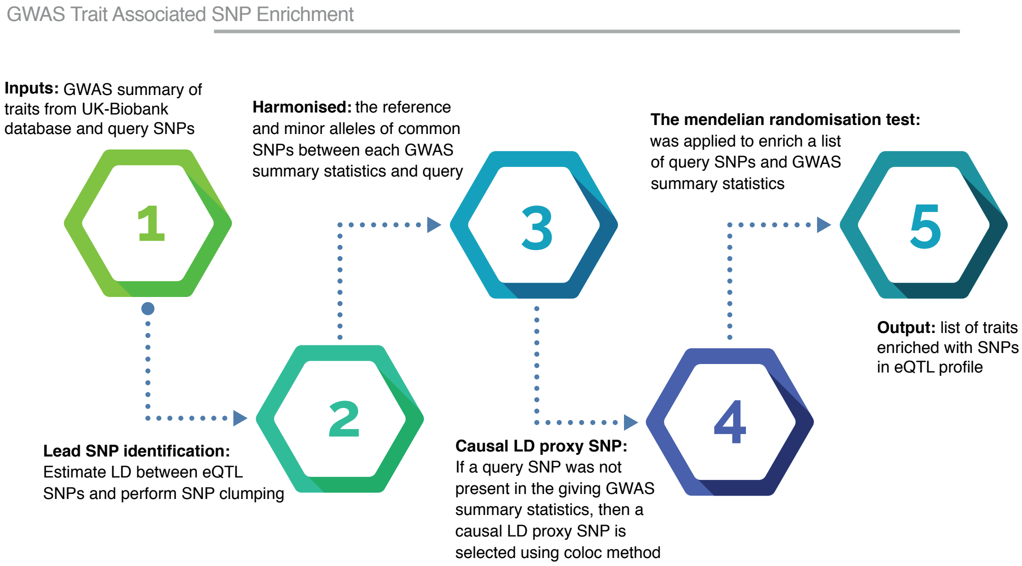

Supplementary figure S11. Integration of eQTL data and GWAS summary statistics using a mendelian randomization-based workflow.
