# Supplementary material for: Genetic determinants of monocyte splicing are enriched for disease susceptibility loci
Source: Nat Commun. 2025 Sep 29;16:8616. doi: 10.1038/s41467-025-63624-7 (PMC12480492; doi:10.1038/s41467-025-63624-7)
Supplement: Supplementary file 2 — Description of Additional Supplementary Tables [file 41467_2025_63624_MOESM2_ESM.pdf]

Description of Additional Supplementary Tables

Supplementary tables are available online on shinyapps.io:
(https://livedataoxford.shinyapps.io/fairfaxlab_supplementary_files/).

Supplementary Data 01: Conditional analysis (QTLtools) to specify context-specific gQTLs and tQTLs across naïve and stimulated monocytes.
Supplementary Data 02: Approximate conditional analysis (moloc) to specify context-specific gQTLs and tQTLs across naïve and stimulated monocytes.
Supplementary Data 03: Replication of significant published scRNA-seq gQTLs in our bulk RNA-seq dataset.
Supplementary Data 04: Summary of results obtained for the causal relationships between g/m/tQTL with GWAS summary statistics in the trait-based analysis in naïve, LPS and IFN-� stimulated monocytes.
Supplementary Data 05: Summary of results obtained for the differential isoform usage in naïve, LPS and IFN-� stimulated monocytes.
Supplementary Data 06: Shared genetic determinants on methylation and gene/transcript expression.
Supplementary Data 07: The allele-specific gene co-expression relationships were found in naïve, LPS and IFN-� analyses (Table 1). The outcomes of functional enrichment analysis of eGens in coExQTLs using curated gene sets from online pathway databases (Table 2).
Supplementary Data 08: The allele-specific gene and methylation site co-expression relationships were found in naïve and LPS analyses.
Supplementary Data 09: The allele-specific transcript co-expression relationships were found in naïve, LPS and IFN-� analyses.
