# Supplementary material for: Genetic determinants of monocyte splicing are enriched for disease susceptibility loci
Source: Nat Commun. 2025 Sep 29;16:8616. doi: 10.1038/s41467-025-63624-7 (PMC12480492; doi:10.1038/s41467-025-63624-7)
Supplement: Supplementary file 12 — Reporting Summary [file 41467_2025_63624_MOESM12_ESM.pdf]

nature portfolio

Corresponding author(s): *Double-anonymous peer review submissions: write DAPR and your manuscript number here instead of author names.*

Last updated by author(s): *YYYY-MM-DD*

Reporting Summary

Nature Portfolio wishes to improve the reproducibility of the work that we publish. This form provides structure for consistency and transparency in reporting. For further information on Nature Portfolio policies, see our Editorial Policies and the Editorial Policy Checklist.

Statistics

For all statistical analyses, confirm that the following items are present in the figure legend, table legend, main text, or Methods section.

n/a	Confirmed	
☐	☒	The exact sample size (*n*) for each experimental group/condition, given as a discrete number and unit of measurement
☐	☒	A statement on whether measurements were taken from distinct samples or whether the same sample was measured repeatedly
☐	☒	The statistical test(s) used AND whether they are one- or two-sided<br>*Only common tests should be described solely by name; describe more complex techniques in the Methods section.*
☐	☒	A description of all covariates tested
☐	☒	A description of any assumptions or corrections, such as tests of normality and adjustment for multiple comparisons
☐	☒	A full description of the statistical parameters including central tendency (e.g. means) or other basic estimates (e.g. regression coefficient) AND variation (e.g. standard deviation) or associated estimates of uncertainty (e.g. confidence intervals)
☐	☒	For null hypothesis testing, the test statistic (e.g. *F*, *t*, *r*) with confidence intervals, effect sizes, degrees of freedom and *P* value noted<br>*Give P values as exact values whenever suitable.*
☐	☒	For Bayesian analysis, information on the choice of priors and Markov chain Monte Carlo settings
☐	☒	For hierarchical and complex designs, identification of the appropriate level for tests and full reporting of outcomes
☐	☒	Estimates of effect sizes (e.g. Cohen's *d*, Pearson's *r*), indicating how they were calculated

Our web collection on statistics for biologists contains articles on many of the points above.

Software and code

Policy information about availability of computer code

Data collection	Manhattan plots of genomic analysis were generated using CMplot (v3.3.3). Local association plots were generated with FUMA (v1.3.5). Box plots and dotplot were generated using ggpubr (v0.2) and customizing ggplot. Visualization of SNPs and methylation data along with annotation as track layers (Lolli plot) was generated using trackViewer (v1.20.2). We used shinyCircos (V2.0) to visualize g/tQTL association as Circos plot126, 127. ChIPseeker package (v1.18.0) was applied to visualize feature distribution and distribution of eSNPs relative to TSS. We leveraged Shiny with R to develop a web application framework on g/tQTL data for programming-free graphical and interactive analysis. The DBI R package was used to execute SQL queries and assigned the results as the input of Shiny (https://livedataoxford.shinyapps.io/ fairfaxlab_supplementary_files/).
Data analysis	We have made a browser available for independent g/tQTL at (https://livedataoxford.shinyapps.io/fairfaxlab_supplementary_files/). The simplified version of the Shiny app is available online on shinyapps.io:<br>gQTLs:<br>https://livedataoxford.shinyapps.io/fairfaxlab_monocytes_eqtl_lps/<br>https://livedataoxford.shinyapps.io/fairfaxlab_monocytes_eqtl_ifn/<br>tQTLs:<br>https://livedataoxford.shinyapps.io/fairfaxlab_monocytes_tqtl_lps/<br>https://livedataoxford.shinyapps.io/fairfaxlab_monocytes_tqtl_ifn/

For manuscripts utilizing custom algorithms or software that are central to the research but not yet described in published literature, software must be made available to editors and reviewers. We strongly encourage code deposition in a community repository (e.g. GitHub). See the Nature Portfolio guidelines for submitting code & software for further information.

Data

Policy information about availability of data

All manuscripts must include a data availability statement. This statement should provide the following information, where applicable:

- Accession codes, unique identifiers, or web links for publicly available datasets
- A description of any restrictions on data availability
- For clinical datasets or third party data, please ensure that the statement adheres to our policy

All sequencing data are made freely available to organizations and researchers to conduct research following the UK Policy Framework for Health and Social Care Research via a data access agreement. Sequence data have been deposited at the European Genome–Phenome Archive, which is hosted by the European Bioinformatics Institute and the Centre for Genomic Regulation under accession no. EGAS00001007111.

Research involving human participants, their data, or biological material

Policy information about studies with human participants or human data. See also policy information about sex, gender (identity/presentation), and sexual orientation and race, ethnicity and racism.

Reporting on sex and gender	Donors for this study were recruited from the Oxford Biobank. Their gender was self-reported and confirmed afterwards as part of the biobank's established protocols.
Reporting on race, ethnicity, or other socially relevant groupings	We clarify in the method section that blood samples were isolated from 192 healthy individuals of European ancestry.
Population characteristics	Our samples are from a healthy biobank (https://www.oxfordbiobank.org.uk) and as such – in keeping with other healthy control cohorts (including the UK biobank) will likely differ in terms of general health (cohort studies tending to have over-representation of those with improved sociodemographics). However, we do not find that there is an association between either age or sex and mitochondria count in these cells, thus any impact of sociodemographic would be expected to minimal compared to the profound effect of exposure to IFN-gamma/LPS and genetics. We have reflected on this point in the manuscript, but we do not use it as covariance to adjust computational models.
Recruitment	Participants who were recruited via the Oxford Biobank (www.oxfordbiobank.org.uk; ethical approval reference 06/Q1605/55), having provided written, informed consent. Volunteers were recruited from the Oxford Biobank.
Ethics oversight	The Oxford Biobank, which facilitated donor recruitment, also approved the study design (www.oxfordbiobank.org.uk; ethical approval reference 06/Q1605/55).

Note that full information on the approval of the study protocol must also be provided in the manuscript.

Field-specific reporting

Please select the one below that is the best fit for your research. If you are not sure, read the appropriate sections before making your selection.

☒ Life sciences ☐ Behavioural & social sciences ☐ Ecological, evolutionary & environmental sciences

For a reference copy of the document with all sections, see nature.com/documents/nr-reporting-summary-flat.pdf

Life sciences study design

All studies must disclose on these points even when the disclosure is negative.

Sample size	Sample size calculation of bulk tissue g/tQTL analysis<br>The powerEQTLSLR R function was utilized to calculate the power for g/tQTL analysis, which involves testing whether a SNP is associated with a gene through simple linear regression (SLR). By supplying values for sample size, minimum detectable slope, standard deviation of the outcome (y) in simple linear regression (sigma.y), and minimum allowable MAF parameters, this function can be utilized to calculate power. Power is used to determine the likelihood of accurately detecting a real association between a genetic variant and gene/transcript expression. If a true association is present, a higher power means a better chance of detecting it.<br>The slope of the simple linear regression parameter from our tQTL/gQTL was adjusted to 0.7 and the MAF was set to 0.04. The estimated testing power for a sample size of 138, with a = 0.2 and family-wise error rate (FWER) = 0.01, was 1.
Data exclusions	The methylation profile of naïve and LPS-stimulated primary monocytes from 176 individuals were assessed using the Illumina 450K array which quantified methylation levels at 300,885 CpG dinucleotides. We excluded 96,427 loci that were analyzed using probes that contained SNP(s) at/near the targeted CpG site (≤ 50 base pair), as these may not be enough to measure DNA methylation levels.
Replication	Comparison and replication of gQTL results<br>We compared gQTL profiles of LPS or IFN-g treated primary monocyte cells formed on microarray profiling with the gQTL formed on RNA-seq profiling of gene expression (P < 0.01).

Randomization	Our samples are from a healthy biobank (https://www.oxfordbiobank.org.uk) and differ in terms of general health (cohort studies tending to have over-representation of those with improved sociodemographics).
Blinding	Given the nature of gQTL analysis, which involves statistical association of genotypes with quantitative traits, blinding was not applicable to the study design or data analysis.

Reporting for specific materials, systems and methods

We require information from authors about some types of materials, experimental systems and methods used in many studies. Here, indicate whether each material, system or method listed is relevant to your study. If you are not sure if a list item applies to your research, read the appropriate section before selecting a response.

Materials & experimental systems

n/a	Involved in the study	
☒	☐	Antibodies
☒	☐	Eukaryotic cell lines
☒	☐	Palaeontology and archaeology
☒	☐	Animals and other organisms
☒	☐	Clinical data
☒	☐	Dual use research of concern
☒	☐	Plants

Methods

n/a	Involved in the study	
☒	☐	ChIP-seq
☒	☐	Flow cytometry
☒	☐	MRI-based neuroimaging

Plants

Seed stocks	NA
Novel plant genotypes	NA
Authentication	NA
